# Attenuation of endoplasmic reticulum stress and mitochondrial injury in kidney with ischemic postconditioning application and trimetazidine treatment

**DOI:** 10.1186/1423-0127-19-71

**Published:** 2012-08-01

**Authors:** Asma Mahfoudh-Boussaid, Mohamed Amine Zaouali, Thierry Hauet, Kaouther Hadj-Ayed, Abdel-Hédi Miled, Sonia Ghoul-Mazgar, Dalila Saidane-Mosbahi, Joan Rosello-Catafau, Hassen Ben Abdennebi

**Affiliations:** 1 Laboratory of human physiology, faculty of pharmacy, university of Monastir, Rue Avicenne, Monastir, 5000, Tunisia; 2 Department of experimental pathology, Hepatic ischemia reperfusion unit, IIBB-CSIC, Barcelona, Spain; 3 Inserm U927, faculty of medicine and pharmacy, university of Poitiers, Poitiers, France; 4 Laboratory of biochemistry, faculty of pharmacy, university of Monastir, Monastir, Tunisia; 5 Laboratory of histology and embryology, faculty of dental medicine, university of Monastir, Monastir, Tunisia

**Keywords:** Kidney, Ischemia-reperfusion, Ischemic postconditioning, Trimetazidine, Endoplasmic reticulum stress, Mitochondria

## Abstract

**Background:**

Endoplasmic reticulum (ER) and mitochondria have been implicated in the pathology of renal ischemia/reperfusion (I/R). In the present study, we investigated whether the use of ischemic postconditioning (IPostC) and trimetazidine (TMZ) separately or combined could reduce ER stress and mitochondria damage after renal ischemia.

**Methods:**

Kidneys of Wistar rats were subjected to 60-min of warm ischemia followed by 120-min of reperfusion (I/R group, n = 6), or to 6 cycles of ischemia/reperfusion (10-s each cycle) just after 60-min of warm ischemia (IPostC group, n = 6), or to i.p. injection of TMZ (3 mg/kg) 30-min before ischemia (TMZ group, n = 6), or to the combination of both treatments (IPostC+TMZ group, n = 6). The results of these experimental groups were compared to those of a sham-operated group in which rat renal pedicles were only dissected. Sodium reabsorption rate, creatinine clearance lactate deshydrogenase (LDH) activity in plasma, and concentration of malonedialdehyde (MDA) in tissue were determined. In addition, Western blot analysis was performed to identify the amounts of cytochrome c, c-JunNH2-terminal kinase (JNK), voltage-dependent anion channel (VDAC), glycogen synthase kinase 3-beta (GSK3-β), and ER stress parameters.

**Results:**

IPostC or/and TMZ significantly decreased cytolysis, oxidative stress and improved renal function in comparison to I/R group. IPostC but not TMZ significantly attenuated ER stress parameters versus I/R group. Indeed, it down-regulated the glucose-regulated protein 78 (GRP78), the activating transcription factor 4 (ATF4), the RNA activated protein kinase (PKR)-like ER kinas (PERK), the X box binding protein-1 (XBP-1) and the caspase12 protein levels. TMZ treatment significantly augmented GSK3-β phosphorylation and reduced levels of cytochrome c and VDAC phosphorylation in comparison to IPostC application. The combination of both treatments gave a synergetic effect. It significantly improved the survival rate, attenuated cytolysis, oxidative stress and improved renal function.

**Conclusion:**

This study revealed that IPostC protects kidney from I/R injury by suppressing ER stress while the beneficial effects of TMZ are mediated by mitochondria protection. The combination of both treatments ameliorated functional recovery.

## Background

Kidney ischemia reperfusion (I/R) injury, which occurs during renal surgery or transplantation, is the major cause of acute renal failure (ARF) [1]. Rapid re-establishment of blood flow is the best way to salvage organ after ischemic injury, but it may also cause reperfusion damage. Growing bodies of research currently concern the possibility of boosting innate protective mechanisms rendering an organ resistant to subsequent injury. For example, ischemic preconditioning (IPC) has been shown to confer protection against a subsequent ischemic attack [2]. IPC is an adaptive response in which brief episodes of ischemia with intermittent reperfusion given immediately before the onset of a more sustained warm ischemia attenuates the pathophysiological effect of I/R injury. In the kidney, IPC has been found to diminish post-ischemic damage [3]. However, a more efficient approach is to intervene at the onset of reperfusion, the timing of which is under the control of the operator. Some results suggest that the first minutes of reperfusion are determinant for defining the final outcome of the kidney after ischemia [1]. In addition, it has been reported that gradual increase of the blood flow to the ischemic kidney could decrease ischemic changes [4]. Therefore, the clinical limitations of IPC have led to the emergence of a new concept i.e. ischemic postconditioning (IPostC), defined as brief intermittent cycles of ischemia alternating with reperfusion applied after the ischemic event.

Ischemic postconditioning is an easy and safe approach, which provides protection against I/R injury in heart [5], brain [6], liver [7] and kidney [1,4]. It has been reported that IPostC can reduce cellular apoptosis [8], attenuate renal dysfunction [1], decrease pro-inflammatory cytokine release after renal I/R [4] and improve kidney graft function [9]. However, the cellular mechanisms underlying the IPostC-induced renal protection against I/R injury remain unclear and considerable research is required for understanding its molecular mechanisms. More recently it has been reported that IPostC protects brain and heart from I/R injury by attenuating endoplasmic reticulum (ER) stress [6,10].

Several studies evidenced the pivotal role of ER stress as a major contributor able to increase the apoptosis and exacerbate cell damage after I/R [11,12]. The ER is a principal site for protein synthesis and folding, calcium (Ca^2+^) storage, and signaling. Alterations in the ER environment as occur during renal I/R lead to the perturbation of Ca^2+^ homeostasis and the accumulation of misfolded proteins [13]. These disorders induce ER stress and in turn activate a well-conserved adaptive response, collectively referred to as unfolded protein response (UPR) [14]. The UPR initially reduces the amount of native proteins and increases the ability of maturation of proteins in the ER. However, when ER stress is intense or prolonged and adaptation mechanisms are overwhelmed, the cells trigger an apoptotic death program [14-16]. The three main mediators involved in the signaling pathway of the ER stress are ATF6 (activated transcription factor 6), IRE1 (inositol requiring enzyme 1) and PERK (protein kinase RNA (PKR)-like ER kinase). Under physiological conditions, these mediators are inactivated by binding to GRP78 in the ER membrane. Initiation of UPR needs their release from GRP78, allowing them to either dimerize or move to other locations within the cell [16].

It became clear that pharmacological agents could also impart protection when administered prior to the onset of a sustained ischemia. Trimetazidine (TMZ), a well-known anti ischemic drug, protects heart [17] and liver [18] against I/R through the activation of intracellular signaling pathways similar to those induced by the IPC. It has also been reported that TMZ protects kidney against the deleterious effects of warm ischemia [19] and reduces the risk of ARF after kidney surgery [20,21]. Besides its metabolic effects, TMZ has been demonstrated to limit oxygen consumption during ischemia, acidosis, intracellular accumulation of sodium and calcium, and thereby maintain the cellular homeostasis [22,23].

The kidney protection conferred by both IPostC and TMZ through suppression of ER stress remains to be defined. The goals of the present study were: (1) to compare the effectiveness of two protective strategies namely IPostC and TMZ when used separately or in combination (2) and to show whether the renal protection conferred by IPostC and TMZ involves suppression of ER stress and mitochondrial impairment in I/R kidney.

## Methods

### Surgical procedure

Male Wistar rats (200–250 g) were anesthetized by ketamine (50 mg/Kg, i.p.) and allowed to breathe spontaneously. This study respected the European Union regulations (Directive 86/609/CEE) for animal experiments. After maintaining the core body temperature at 37°C, a midline laparatomy was performed, and both left and right renal arteries and veins were exposed. In order to evaluate kidney function, catheters were inserted into ***i.*** the jugular vein to perfuse (Minipuls 3 peristaltic pump, Gilson, France) heated (37°C) mannitol (10%) and heparin (50 U/I); ***ii.*** the carotid artery to measure arterial pressure (Pression Monitor BP-1; Pression Instruments, Sarasota, FL) and to collect blood samples collection ; ***iii.*** the bladder to collect urine samples. To induce renal ischemia the renal pedicles were occluded with non traumatic vascular clamps. Reperfusion was initiated by removal of the clamps.

### Experimental protocol

**Figure 1:**
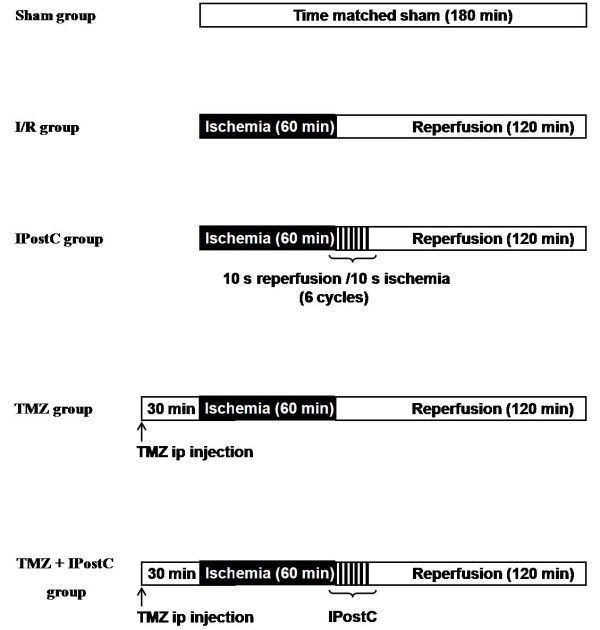
**Experimental protocol.** A schematic drawing of the experimental protocol used to determine the effect of ischemic postconditioning (IPostC, 6 cycles of 10-s of reperfusion and 10-s re-occlusion, application after ischemia), and the effect of trimetazidine (TMZ, 3 mg/kg, i.p. injection 30-min before ischemia). Sham, sham-operated rats; I/R, rats were subjected to bilateral renal ischemia (60 min) followed by reperfusion (120 min).

Rats were randomly divided into 5 experimental groups (n = 6 for each group, Figure 1):

Sham-operated control group: kidney pedicles were only dissected off the surrounding perirenal fat;

Ischemia/reperfusion (I/R) group: both renal pedicles were clamped for 60 min and then subjected to 120 min of reperfusion;

Ischemic postconditioning (IPostC) group: as I/R group but kidneys were subjected to 6 cycles of 10-s of reperfusion and 10-s re-occlusion immediately after the 60-min of ischemia;

Trimetazidine (TMZ) group: as I/R group but rats were treated with 3 mg/Kg (i.p.injection) of TMZ 30-min before warm ischemia;

TMZ+IPostC group: 30-min after TMZ injection (3 mg/Kg, i.p.), renal pedicles were clamped for 60-min and then IPostC (6 cycles of 10 sec I/R) was applied.

### Survival rates

Another 30 rats were randomly divided into 5 groups for observation of survival rates during 10 days after reperfusion. After reperfusion, the animals were returned to their cages and were allowed for food and water as needed.

### Renal function study

Blood and urine samples were collected to assess creatinine and sodium concentration. Creatinine concentrations in plasma and urine were measured according to the Jaffe’s reaction (BioMerieux Kit, France). Sodium concentrations in plasma and urine were evaluated by a flame photometer (BT.634, Biotecnica instruments, Italy).

Renal glomerular function was assessed by creatinine clearance. The formula to calculate the creatinine clearance (μl/min/g BW) was: $\left({\text{Creat}}_{\text{u}}.\text{ V}\right)/{\text{Creat}}_{\text{p}}$

Creat_p_: Creatinine concentration in plasma (μmol/l).

Creat_u_: Creatinine concentration in urine (μmol/l).

V: Urine flow (μl/min).

Renal tubules function was evaluated by sodium reabsorption rate. The formula to calculate the sodium reabsorption rate (%) was: $100-\left[100.\left({\text{Na}}_{\text{u}}.{\text{ Creat}}_{\text{p}}\right)/{\text{Na}}_{\text{p}}.{\text{ Creat}}_{\text{u}}\right]$

Na_u_: Sodium concentration in urine (mmol/l).

Na_p_: Sodium concentration in plasma (mmol/l).

Créat_p_: Creatinine concentration in plasma (μmol/l).

Créat_u_: Creatinine concentration in urine (μmol/l).

### Lipid peroxydation assessment

Lipid peroxydation, used as an indirect index of the oxidative injury induced by the reactive oxygen species, was determined by measuring the formation of MDA with the thiobarbiturate reaction [24].

### Histological examination

To appraise the severity of kidney injury, tissues sections were stained with hematoxylin and eosin and evaluated by an experienced pathologist who was unaware of the treatment. A grading scale of 0–4, as outlined by Jablonski et *al.*, was used for the histopathologic assessment of kidney damage [25].

### Western blot analysis

The kidney tissue was homogenized in ice cold lysis buffer (containing 150 mM NaCl, 50 mM Tris–HCl (pH 7.5), 1 mM DTT, 50 mM NaF, 1 mM PMSF, 1 mM EDTA, 1 mM EGTA, 0.1 mM orthovanadate, 0.05% Triton-X 100 and 2% protease inhibitor cocktails) using a Teflon homogenizer. After that, the homogenates were centrifuged at 15000 rpm for 20 min at 4°C. The supernatants were collected to determinate protein concentrations by the Bradford assay. Proteins were then separated by SDS-PAGE electrophoresis and transferred into polyvinyldene fluoride membranes. Membranes were immunoblotted with antibodies directed against GRP78, XBP-1, total and phospho-PERK and ATF4 (Santa Cruz Biotechnology, Santa Cruz, CA, USA), caspase 12, cytochrome c, total and p-GSK3-β, total and p-VDAC and total and phospho-JNK (Cell Signaling Technology Inc., Beverly, MA, USA) and β actin (Sigma Chemical, St. Louis, MO). The bands were visualized using an enhanced chemiluminescence kit (Bio-Rad Laboratories, Hercules, CA, USA). The values were obtained by densitometric scanning and the quantity one software program (Bio-Rad Laboratories, Hercules, CA, USA).

### Statistical analysis

All data were expressed as mean values ± SEM (n = 6 for each group). The one-way analysis of variance (ANOVA) test was used for comparison of group data. If the ANOVA was significant, multiple comparisons were carried out using the Newman-Keuls test. Statistical significance was defined as P <0.05. Survival curves were constructed using the khi 2 test differences between the four groups were analyzed by the log-rank test. A P value <0.05 was considered a statistical significance.

## Results

### Effects of the application of IPostC and ipTMZ treatments separately

IPostC and TMZ treatments improved survival rates when compared to I/R group. We found respectively 83% and 67% in IPostC and TMZ groups versus 33% in I/R group. Sham-operated rats showed no mortality (survival rate was 100%).

**Figure 2:**
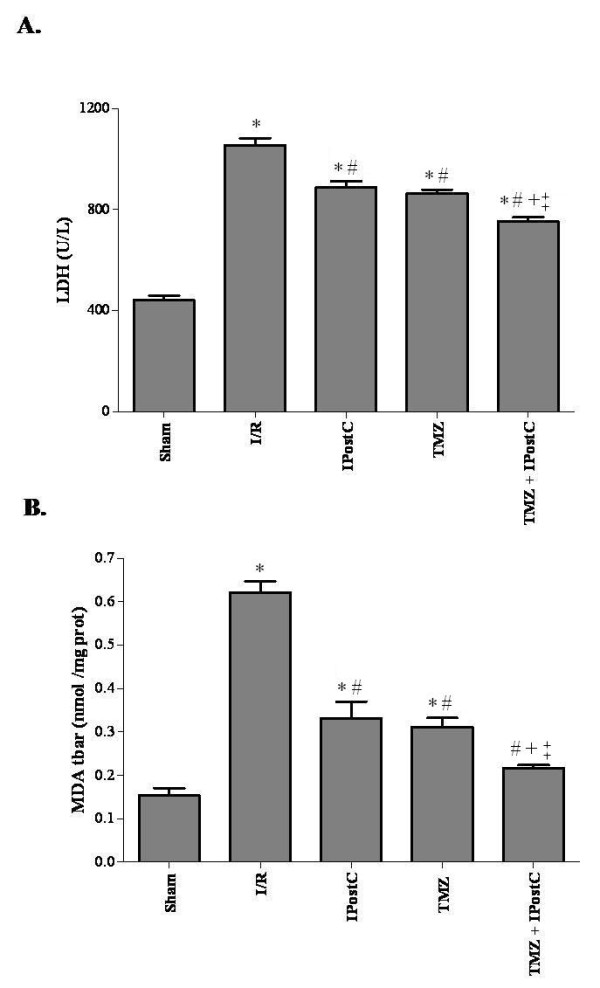
**Evaluation of lactate dehydrogenase activity in plasma (A) and malonedialdehyde concentration in tissue (B).** Results are presented as mean ± SEM (n = 6 in each group). ^*^P <0.05 vs. Sham group. ^#^*P <*0.05 vs. I/R group. ^+^*P <*0.05 vs. IPostC group. ^‡^*P <*0.05 vs. TMZ group.

Renal I/R resulted in a significant (P <0.05) increase in plasma LDH activity (1054 ± 28 U/L, Figure 2A) and tissue MDA concentration (0.62 ± 0.02 nmol/mg prot, Figure 2B) compared to sham group (441 ± 18 U/L for LDH activity and 0.15 ± 0.01 nmol/mg prot for MDA concentration). The use of IPostC or TMZ treatments significantly improved cells integrity and reduced lipid peroxidation when compared to I/R group. Indeed after IPostC application, we observed 888 ± 23 U/L for LDH activity and 0.33 ± 0.03 nmol/mg prot for MDA concentration (P <0.05 vs I/R group, respectively). After i.p. injection of TMZ, we found that LDH activity decreased to 864 ± 14 U/L and tissue MDA concentration reached 0.31 ± 0.02 nmol/mg prot P <0.05 vs I/R group, respectively. There is no statistical difference between TMZ and IPostC groups.

**Figure 3:**
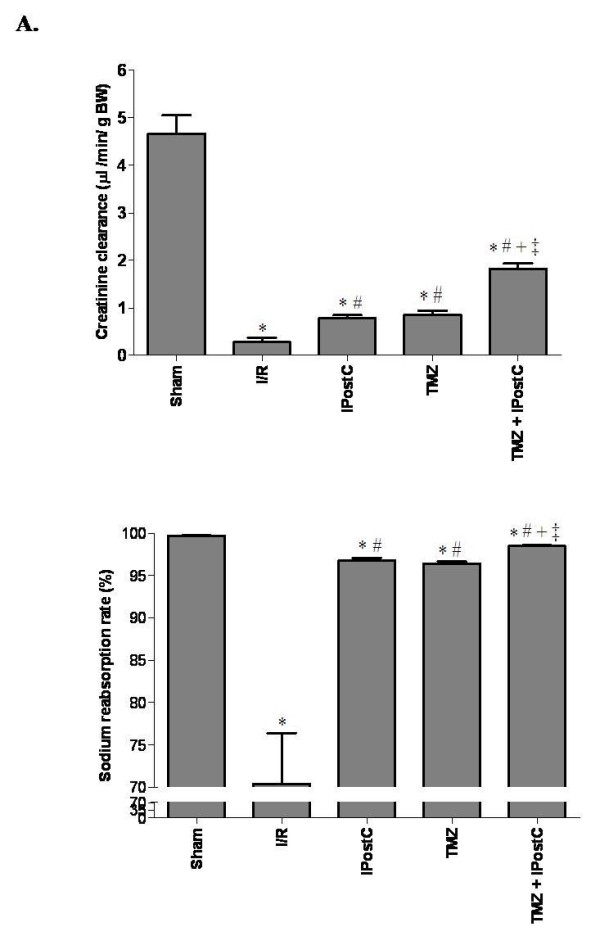
**Evaluation of creatinine clearance (A) and sodium reabsorption rate (B).** Results are presented as mean ± SEM (n = 6 in each group). ^*^P <0.05 vs. Sham group. ^#^*P <*0.05 vs. I/R group. ^+^*P <*0.05 vs. IPostC group. ^‡^*P <*0.05 vs. TMZ group.

Kidney function parameters (Figure 3) of rats subjected to I/R were also significantly different from those of sham-operated group. Indeed, I/R decreased renal creatinine clearance (0.27 ± 0.09 vs 4.66 ± 0.4 μL/min/g BW, for I/R and sham groups respectively, P <0.05) and sodium reabsorption rate (70.4 ± 6.0 vs 99.7 ± 0.02%, for I/R and sham groups respectively, P <0.05). Both IPostC and TMZ treatments significantly improved function parameters of the ischemic kidneys. When IPostC and TMZ were applied, we found respectively 0.78 ± 0.06 and 0.85 ± 0.09 μL/min/g BW for creatinine clearance (P <0.05 vs I/R group, respectively) and 96.8 ± 0.3% and 96.4 ± 0.3% for the sodium reabsorption rate (P <0.05 vs I/R group, respectively). There is no statistical difference between IPostC and TMZ groups for both parameters.

**Figure 4:**
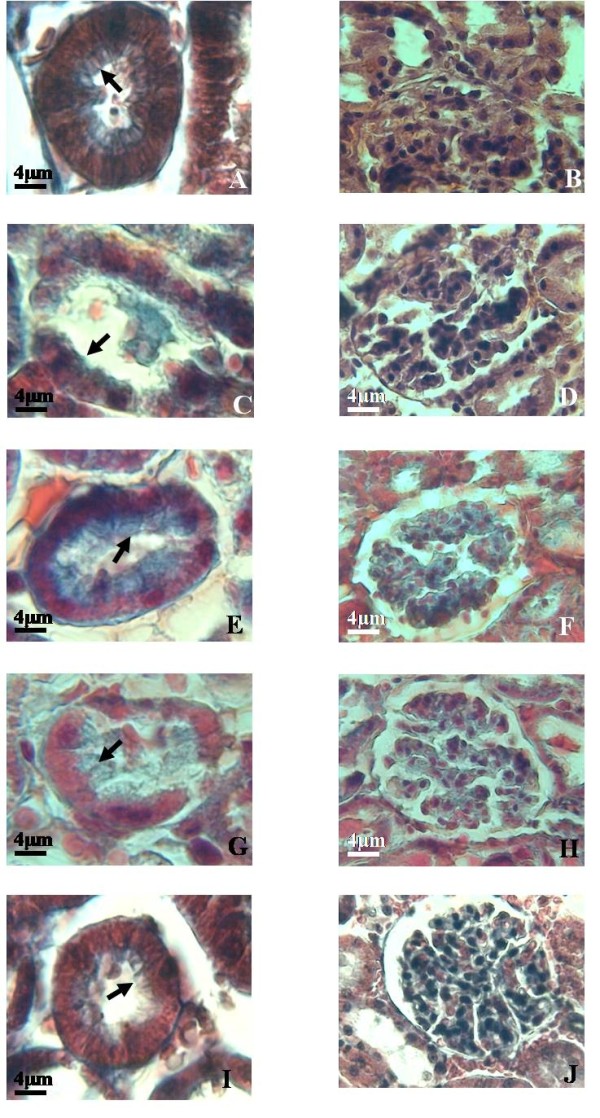
**Histologic examinations of renal tissues.** Sham group (A&B), I/R group (C&D), IPostC group (E&F), TMZ group (G&H) and TMZ+IPostC group (I&J). Arrow tips indicate brush border. I/R induced loss of brush border, but IPostC or/and TMZ preserve brush border. Magnification, x200.

**Figure 5:**
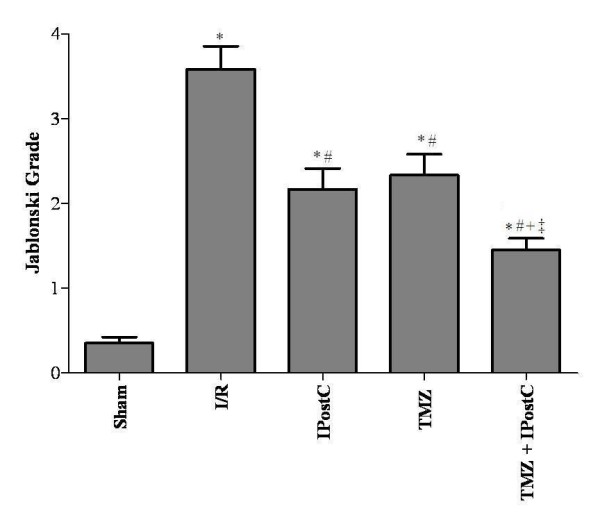
**Jablonski scores.** Histologic assessment was performed by an experimented renal pathologist who was unaware of the treatments. A minimum of 8 fields for each slide were examined. The following histologic features were observed: interstitial edema, epithelial flattening, loss nuclei, fragmented luminal cells/casts, proteinaceous casts. Scores are presented as mean ± SEM (n = 6 in each group). ^*^P <0.05 vs. Sham group. ^#^*P <*0.05 vs. I/R group. ^+^*P <*0.05 vs. IPostC group. ^‡^*P <*0.05 vs. TMZ group.

Renal I/R caused severe architectural disruption, including tubular necrosis, medullary hemorrhage, congestion and development of proteinaceous casts (Figure 4C and 4D). According to Jablonski scores (Figure 5), 60 min renal ischemia followed by 120 min reperfusion resulted in severe kidney injury. In contrast, IPostC (Figure 4E and 4F) and TMZ (Figure 4G and 4H) preserved the morphologic integrity of tubular structure and tubular brush border with less evidence of acute tubular necrosis and less casts formation. Quantitative analysis (Figure 5) showed a decreased score in IPostC and TMZ groups compared with I/R group (P <0.05, respectively).

**Figure 6:**
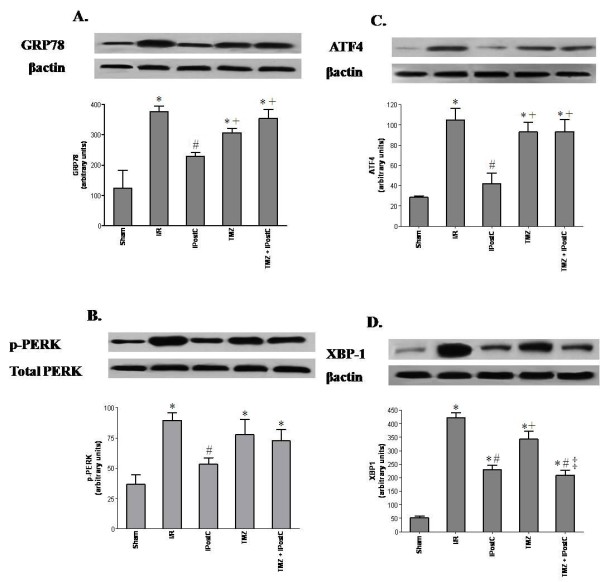
**Western blot of GRP78 (A), total and phosphorylated-PERK (B), ATF4 (C) and XBP-1 (D) protein levels.** The upper panels show one representative blot of six independent experiments and the lower panels show densitometric evaluation of the independent Western blot. Results are presented as mean ± SEM (n = 6 in each group). ^*^P <0.05 vs. Sham group. ^#^*P <*0.05 vs. I/R group. ^+^*P <*0.05 vs. IPostC group. ^‡^*P <*0.05 vs. TMZ group.

We also considered ER stress. As indicated in Figure 6, our results showed a significant (P <0.05) enhance in GRP78, p-PERK, ATF4 and XBP-1 protein levels after I/R in comparison to sham-operated group, which denotes ER stress. The IPostC application before reperfusion of ischemic kidneys significantly reduced GRP78 level (228 ± 13 vs 376 ± 18 for IPostC and I/R groups respectively, P <0.05). Similarly, this treatment significantly declined the relative amounts of p-PERK (53 ± 5 vs 89 ± 6, P <0.05), ATF4 (42 ± 11 vs 105 ± 12, P <0.05) and XBP-1 (231 ± 16 vs 423 ± 19, P <0.05) compared to I/R, respectively. Interestingly, no statistical differences were obtained between sham and IPostC groups for all these parameters, except for XBP-1. In contrast, i.p. injection of TMZ before warm ischemia did not improved ER stress parameters versus I/R group which remained high compared to sham group (P <0.05).

**Figure 7:**
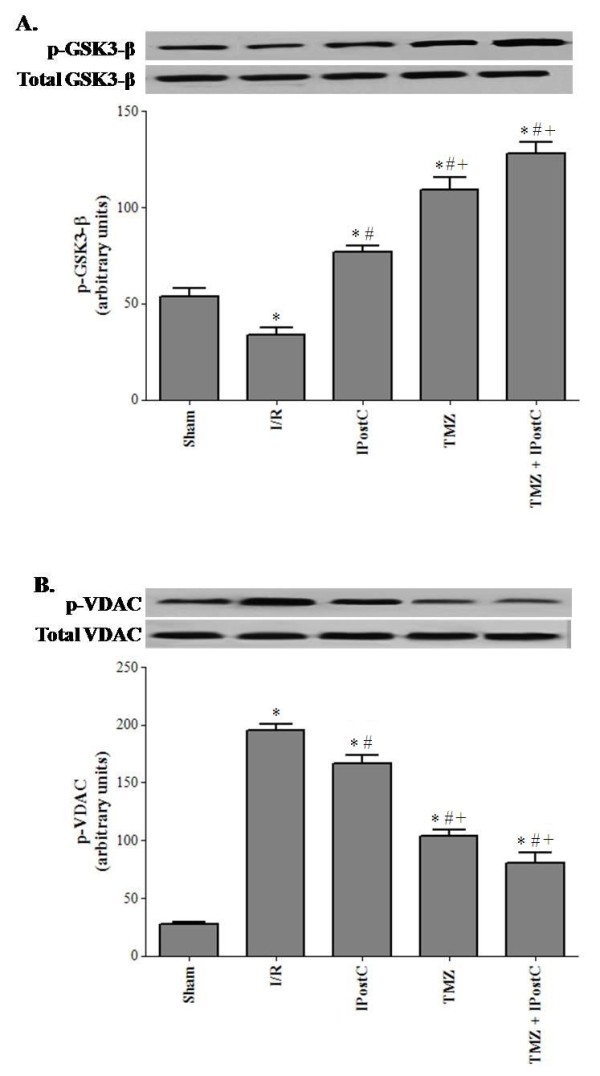
**Western blot of total and phosphorylated-GSK3-β (A), and total and phosphorylated-VDAC (B) protein levels.** The upper panels show one representative blot of six independent experiments and the lower panels show densitometric evaluation of the independent Western blot. Results are presented as mean ± SEM (n = 6 in each group). ^*^P <0.05 vs. Sham group^. **#**^*P <*0.05 vs. I/R group. ^+^*P <*0.05 vs. IPostC group.

Considering the potential relationship between GSK3_−_β and mitochondrial VDAC, we evaluated whether changes in GSK3-β activity induced by ischemic injury could be associated with changes in mitochondrial VDAC activity (Figure 7). The Western blot analysis of ischemic kidneys revealed a decline of GSK3-β phosphorylation (34 ± 4 vs 54 ± 4, P <0.05) and an increase of VDAC phosphorylation (195 ± 6 vs 27 ± 2, P <0.05) in comparison to sham, respectively. Of the two treatments used, TMZ affected more p-GSK3-β and p-VDAC. Indeed, we found 109 ± 7 and 104 ± 2 for respectively p-GSK3-β and p-VDAC in the TMZ group (P <0.05 vs I/R respectively), and 77 ± 3 and 167 ± 7 for respectively p-GSK3-β and p-VDAC in the IPostC group.

**Figure 8:**
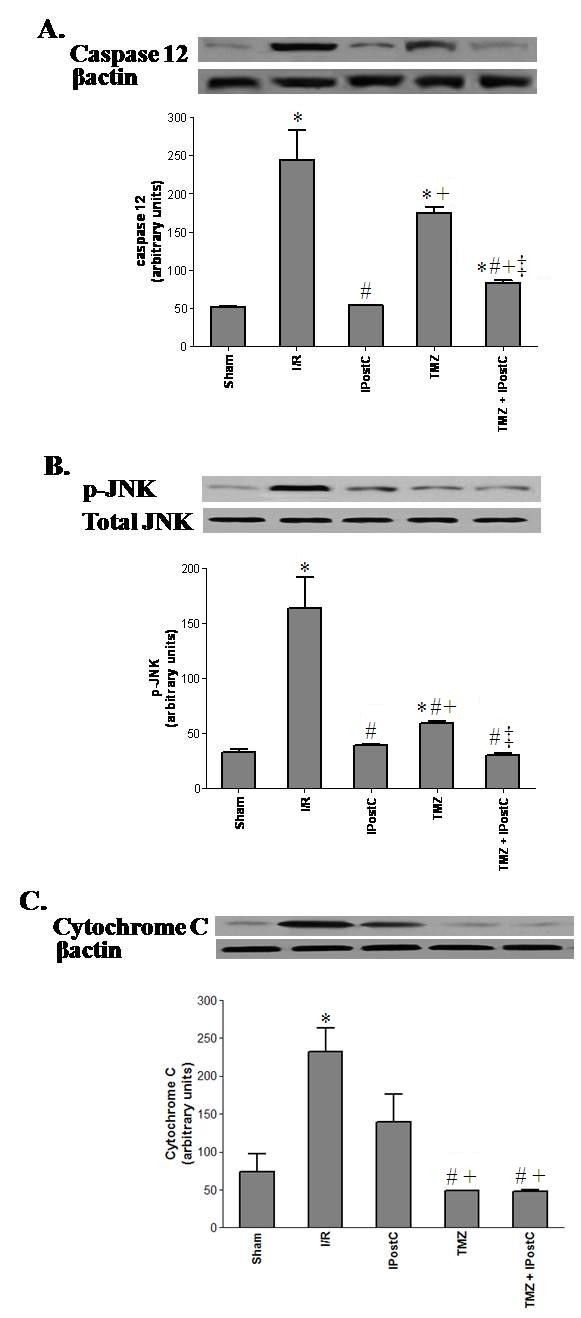
**Western blot of cleaved caspase 12 (A), total and phosphorylated-JNK (B) and cytochrome c (C) protein levels.** The upper panels show one representative blot of six independent experiments and the lower panels show densitometric evaluation of the independent Western blot. Results are presented as mean ± SEM (n = 6 in each group). ^*^P <0.05 vs. Sham group. ^#^*P <*0.05 vs. I/R group. ^+^*P <*0.05 vs. IPostC group. ^‡^*P <*0.05 vs. TMZ group.

The relevance of apoptosis was assessed by tissue caspase 12, p-JNK, and cytochrome c protein levels (Figure 8). Compared to sham group, renal I/R resulted in a significant increase in protein levels of caspase 12 (245 ± 38 vs 52 ± 1, P <0.05), p-JNK (163 ± 28 vs 32 ± 1, P <0.05) and cytochrome c (232 ± 31 vs 74 ± 23, P <0.05). Both treatments induced distinct results on the apoptosis parameters. Indeed in comparison to I/R, IPostC application attenuated caspase 12 and p-JNK (54 ± 1, P <0.05 and 39 ± 1, P <0.05 respectively) whereas TMZ injection declined only cytochrome c release (49 ± 1, P <0.05 vs I/R group).

### Effects of the combination of TMZ and IPostC treatments

Rats treated with TMZ+IPostC had significantly (P <0.05) prolonged survival rate (100%) compared with those in I/R (33%), IPostC (83%) and TMZ (67%). In addition, combination of TMZ and IPostC treatments improved cells integrity and reduced lipid peroxidation when compared to each treatment separately. Indeed, LDH activity and tissue MDA concentration reached 753 ± 16 U/L and 0.21 ± 0.01 nmol/mg prot, respectively (P <0.05 vs TMZ and IPostC groups, respectively). Parallely, creatinine clearance of the kidneys was also increased. We found 1.82 ± 0.1 μL/min/g BW, it was significantly (P <0.05) improved when compared to TMZ and IPostC groups, respectively. The combination of these two treatments preserved the morphologic integrity of tubular structure (Figure 4I and 4J). Quantitative analysis showed a further synergistic effect in decreasing Jablonski scores as compared to each treatment alone (P <0.05 versus IPostC and TMZ respectively).

Concerning mitochondrial and apoptosis parameters, the beneficial effects were maintained when both treatments were combined. In fact, we found 125 ± 7 and 80 ± 3 respectively for GSK3-β and VDAC (P <0.05 vs IPostC group, respectively). However, these improvements were not observed regarding all ER stress parameters; expect the XBP-1 factor.

## Discussion

Blood flow restoration next an anoxic or hypoxic period alters the function of numerous organs, such as the heart [2], brain [6], liver [18] and kidney [4]. This event induces abnormal activation of many signal transduction cascades, which lead to cellular dysfunction and initiation of the apoptosis/necrosis. In agreement with previous studies [3,26], our results confirmed that renal I/R provokes severe glomerular and tubular damage. Excessive production of reactive oxygen species (ROS) plays a principal role leading to structural and functional injury of the ischemic kidneys [27].

The understanding of renal I/R syndrome is still incomplete although several mechanisms were henceforth defined. Some recent evidences strongly suggest the involvement of ER stress as an initiator of cell death during ischemic glomerular and tubular epithelial injury [28,29]. The ER provides an optimized environment for formation and maturation of cell proteins, but several insults can interfere with this machinery causing aberrant protein folding. Accumulation of these unfolded or misfolded proteins in the ER lumen induces ER stress and in turn activates a well-conserved adaptive response.

The present study shows that the ER stress is enhanced in kidney following I/R. We observed increased amount of the ER chaperone GRP78, which is a central regulator of ER function. This expression was associated with amplified levels of p-PERK, ATF4 and XBP-1. In addition, apoptosis is induced given that caspase 12 and p-JNK, the hallmarks of ER associated apoptosis, and cytochrome c were found to be over-expressed in ischemic kidney. Other studies have also produced evidence suggesting ER stress following *in vivo* renal I/R. I/R induces phosphorylation of PERK and eIF2α, indicating ER stress and activation of the UPR [28,30,31]. It has been observed that I/R of rat kidneys augmented expression of the ER molecular stress chaperone GRP78, as well as TUNEL labeling and the apoptotic protein caspase 12. These increases are concomitant with oxidative stress [29]. In their study, Wang et *al.* have established that one of the downstream pathways of apoptotic cell death after ER stress is mediated by JNK [32]. They found that JNK is translocated to the mitochondrial membrane, and in turn it is decisive for cytochrome c release. Furthermore, the increased level of p-PERK after I/R injury seems to play a significant pro-apoptotic role, as it induces the activation of the CCAAT/enhancer-binding protein homologous protein (CHOP) [31]. Gao et *al.* showed that taurousodeoxycholic acid (TDUCA), an inhibitor of ER stress, could represent effective strategy to improve renal function and to reduce apoptosis of tubular epithelial cells. These beneficial effects were associated with a reduction of GRP78, CHOP and caspase 12 [33]. Taken together, these data and our results suggest that I/R-induced oxidative injury activates ER stress and consequently leads to damage cells of rat kidneys. However, it is unclear how ROS induce ER stress. One possibility is that ROS cause inhibition of Ca^2+^-ATPase on the ER membrane and thus depletion of Ca^2+^ stores in this organelle [13,34,35]. Another possibility is that ROS may cause generation and accumulation of oxidatively modified and abnormal proteins in the ER [36].

The detrimental effects of renal I/R injury are now well recognized. Interestingly, IPostC has been shown to protect multiple organs [5-7] including kidney [8] from I/ R injury. Chen et *al.* reported that IPostC application attenuated renal dysfunction [4]. In agreement with this our results clearly indicate an improvement of creatinine clearance and Na reabsorption rate in the treated kidneys versus those none treated. Parallely, IPostC enhances cell integrity and decreases lipid peroxydation as compared to I/R group. Eldaif et *al.* demonstrated that IPostC could stabilize the structure of the tubular epithelial cells [9]. However, the cellular mechanism remains to be defined. Our results show a marked decline in the levels of GRP78, p-PERK, ATF4, and XBP-1 in IPostC group. Concomitantly, we note that IPostC reduces the phosphorylation of the JNK and the caspase 12 level in ischemic kidneys. Yeh et *al.* observed that renal hypoxic conditioning attenuated oxidative stress, and diminished GRP78 and caspase 12 in the ischemic renal tubules. These effects are correlated with enhanced HSP70 protein expression and hypoxia-inducible transcription factor-1α (HIF-1α) activation [29]. In their study, Liu et *al.* confirmed that IPostC reduced I/R induced ER stress in the heart via the mobilization of the p38 mitogen-activated protein kinase (p38 MAPK)/JNK signaling pathways [10]. Moreover, Yuan et *al*. suggested in their study that IPostC protected ischemic brain through suppressing ER stress-induced apoptosis and that PI3K/Akt pathway was involved [6]. Therefore we could suggest that IPostC could protect kidneys against renal I/R insults throughout modulation of ER stress.

There is accumulating evidence that lethal reperfusion injury could be reduced by TMZ preconditioning in animals. It was found that TMZ conserves ATP production and lowers intracellular acidosis, while maintaining cellular homeostasis [19-21,37]. In addition, it reduces oxidative damage to the mitochondria and protects the organ from I/R-induced damage arising from mitochondrial respiration [22,38,39]. When given before ischemia, TMZ attenuates the inflammatory response and the tubulointerstitial development of fibrosis prevalent in ischemic kidney injury and reduces the rate of apoptosis expression [19,22]. These effects were correlated with an early and great expression of HIF-1α. The same impact was also found in liver [18]. In another study, the protective effect appeared to be through activation of p38 MAPK and Akt signaling [17]. However, there is no data about the impact of TMZ treatment on ER stress processes after I/R. Here, we found that contrary to IPostC treatment, it seems that reduction of ER stress is not involved in the protective mechanism of TMZ of ischemic kidney, at least at the early period of reperfusion. As observed, all levels of the ER stress parameters (GRP78, p-PERK, ATF4, XBP-1 and caspase 12) were decreased but not significantly as compared with those of I/R group. However, we noted a significant decrease of mitochondrial parameters (p-VDAC and cytochrome c) and a significant increase in p-GSK3-β content in TMZ group versus I/R group.

TMZ as mostly described, acts on mitochondria by restoring ATP synthesis and by maintaining the mitochondrial membrane impermeability [40]. Indeed, TMZ might bind to inner mitochondrial membrane [41] to modulate the mitochondrial permeability transition pore (mPTP) opening [19]. Moreover, TMZ might interact with VDAC which has been also proposed to control the release of cytochrome c without mPTP opening [41]. Parallely, previous studies demonstrated that inhibition of GSK3-β phosphorylation was associated with a decrease in VDAC phosphorylation and a delayed mPTP opening [19,42,43].

Overall, the data from the present study suggest that ER might not be a target through which TMZ exerts its cytoprotective effect at early reperfusion and that TMZ beneficial effect attenuated essentially mitochondrial dysfunction after renal I/R. In agreement with this, our results show that TMZ protected mitochondria from deleterious consequences of renal I/R injury, via promotion of GSK3-β phosphorylation and prevention of VDAC phosphorylation.

We also evaluated the effect of the combination of both TMZ and IPostC treatments on renal function. Interestingly, we noted a significant improvement of the renal functional parameters as shown by a rise of GFR and Na reabsorption rate versus TMZ and IPostC separately. Such result may be explicated, at least partially, by the improvement of structural parameters. In fact, we observed a significant decline of MDA level and LDH activity as compared to each treatment alone. Histopathological assessment confirmed our suggestion. Taken together, our results supported the idea of a synergetic outcome of both treatments to ameliorate kidney function following warm I/R. The protective mechanisms of the association TMZ+IPostC implicate distinct organelles. In fact, the beneficial effects of TMZ triggered essentially mitochondrial disturbance, while IPostC reduced ER stress. We noted, after the association of both treatments, a significant decline in the rate of XBP-1 and caspase 12, as compared to the TMZ group and I/R group. However, we noted a paradoxical increase in the levels of the PERK and its downstream factor (ATF4) in the group TMZ+IPostC, as compared to IPostC only. These results may be explained, by the fact that the combination TMZ+IPostC did not implicate the PERK pathway and its downstream factor (ATF4). Probably, the effect of IPostC on the PERK pathway is lost, when it was combined to TMZ treatment. The underlying mechanisms are not well understood and other investigations are necessary to confirm this hypothesis.

## Conclusions

I/R injury is a multifactorial process in which mitochondria and ER take critical part. Consequently, treatments that could act on these organelles represent interesting preventive strategies. This study demonstrates that application of IPostC after warm ischemia or i.p. injection of TMZ before warm ischemia attenuates renal dysfunction and renal morphologic disruptions during early reperfusion. TMZ acts on mitochondria while the use of IPostC seems to trigger mechanisms which are associated, with an attenuation of ER dysfunction. The combination of these treatments appears to give a synergetic effect on functional level.

## Abbreviations

ARF, Acute renal failure; ATF4, Activating transcription factor 4; CHOP, CCAAT/enhancer-binding protein homologous protein; ER, Endoplasmic reticulum; GRP78, Glucose-regulated protein 78; GSK3-β, Glycogen synthase kinase 3-beta; HIF-1α, Hypoxia-inducible transcription factor-1α; I/R, Ischemia reperfusion; IPC, Ischemic preconditioning; IPostC, Ischemic postconditioning; JNK, c-JunNH2-terminal kinase; LDH, Lactate dehydrogenase; MDA, Malonedialdehyde; mPTP, Mitochondrial permeability transition pore; PERK, RNA activated protein kinase (PKR)-like ER kinase; p38 MAPK, p38 mitogen-activated protein kinases; ROS, Reactives oxygen species; TDUCA, Taurousodeoxycholic acid; TMZ, Trimetazidine; UPR, Unfolded protein response; VDAC, Voltage-dependent anion channel; XBP-1, X box binding protein-1.

## Competing interests

The authors declare that they have no competing interests.

## Authors’ contributions

AMB and MAZ carried out the experimental work and analyzed data. AMB, HBA and JRC designed the study, coordinated the experiments, analyzed data and wrote the manuscript. KHA conducted the statistical analyses. AHM and SGM conducted the biochemical analyses. DSM and TH analyzed the data. All authors read and approved the final manuscript.
